# Galectin-3 in AKI: Pathogenic Target, Diagnostic Biomarker, or Both?

**DOI:** 10.7150/ijbs.129063

**Published:** 2026-01-29

**Authors:** Yuan Gui, Dong Zhou

**Affiliations:** Division of Nephrology, Department of Medicine, University of Connecticut, School of Medicine, Farmington, CT, USA, 06030.

Acute kidney injury (AKI) remains one of nephrology's most unpredictable and clinically challenging syndromes, largely because its molecular drivers differ widely across insults such as ischemia, toxins, and sepsis. Yet at the bedside, clinicians continue to rely on serum creatinine and urine output, parameters relatively lag behind actual epithelial injury and are easily influenced by hemodynamics, medications, or systemic stress. Creatinine rises only after substantial nephron loss, while urine output can be influenced by non-renal factors. These limitations point to the ongoing need for biomarkers that detect injury early and provide insight into its molecular basis.

The past decade has witnessed intense efforts to identify early and reliable biomarkers capable of capturing tubular stress before functional decline. Molecules such as kidney injury molecule 1 (KIM1), neutrophil gelatinase-associated lipocalin (NGAL), and matrix metalloproteinase-7 (MMP-7) have demonstrated early diagnostic values^1^-^3^. For example, a multi-center prospective cohort revealed that urinary MMP-7 peaked within six hours in patients who developed severe AKI after cardiac surgery and remained elevated for 48 hours, whereas serum creatinine peaked more than 24 hours later^2^. Such findings reflect the feasibility of tracking tubular response in real time after AKI. In this issue, *Liu et al.* advanced this concept by revealing that Galectin-3, long associated with immune cell activation, in fact represented a convergent epithelial injury program intrinsically activated within proximal tubules across diverse AKI models. Through elegant multi-model and multi-omics integration, they outlined a unified mechanistic pathway in which Krüppel-like factor 4 (KLF4) drove Galectin-3 expression to promote tubular apoptosis and amplify inflammatory signaling^4^.

A major strength of their study lay in its breadth and integrative depth. By harmonizing single-cell transcriptomic datasets across four distinct AKI models, *Liu et al*. distinguished insult-specific responses from a shared epithelial injury signature. Galectin-3 emerged as one of the most consistently upregulated genes in injured or failed-repaired proximal tubules^4^, a finding previously obscured by its canonical association with immune cells and systemic inflammation. Their demonstration that Galectin-3 was strongly induced within tubules reframes it as a key epithelial effector rather than merely an inflammation-associated bystander, although in chronic kidney disease its expression in tubular, endothelial, and immune cells has been linked to adverse outcomes^5^.

Human data further strengthened its translational appeal. Tubular Galectin-3 expression positively correlated with histologic injury severity, and urinary Galectin-3 levels were substantially elevated in AKI patients^4^. Unlike serum creatinine, which reflects filtration rather than injury, Galectin-3 more directly captures epithelial perturbation. Its mechanistic proximity to damage and detectability in urine positioned it as a promising non-invasive biomarker capable of reporting epithelial stress, stratifying severity, or potentially identifying at-risk patients before functional decline becomes evident. Looking ahead, integrating Galectin-3 with established stress biomarkers such as KIM-1, NGAL, MMP-7, and [TIMP2]x[IGFBP7] could enhance risk stratification by capturing the full spectrum of epithelial injury, from early tubular cell stress to subsequent cell death and inflammatory responses. Additional clinical studies also supported its systemic relevance, for instance, ICU patients with AKI exhibited persistently elevated serum Galectin-3 at discharge^6^, particularly in sepsis-associated AKI and in non-survivors^7^.

Mechanistically, *Liu et al* placed KLF4 at the center of Galectin-3 induction in tubular cells. KLF4 bound the *Lgals3* promoter and drove its transcription^4^, but in a cell-type-specific manner that contrasts sharply with KLF4's protective roles in podocytes^8^, endothelial cells^9^, and macrophages^10^. In proximal tubules, KLF4 functioned as a stress-induced activator of Galectin-3, triggering apoptotic and inflammatory cascades during cisplatin- or ischemia-induced damage. This divergence highlights the complexity of kidney transcriptional regulation and raises a provocative therapeutic question: is Galectin-3 a tractable pathogenic target?

The pharmacological and genetic evidence from *Liu et al*'s study argues yes. Using GB1107, a selective Galectin-3 inhibitor, they showed that blocking Galectin-3 signaling preserved kidney function and reduced tubular cell death after cisplatin exposure^4^. Tubule-specific *KLF4* knockout mice exhibited similar protection from cisplatin- and ischemia-induced AKI, with reduced apoptosis, attenuated structural damage, lower injury marker expression, and diminished immune cell infiltration^4^. These findings firmly establish the KLF4/Galectin-3 axis as an active driver of epithelial injury rather than a passive biomarker. Therapeutically, the ability to modulate a defined epithelial injury program represents a meaningful advance in a clinical landscape with few mechanism-based AKI interventions.

Nevertheless, several limitations warrant consideration. First, although Galectin-3 was consistently upregulated across AKI models, its distinct functions in different kidney cell types, including proximal tubules, macrophages, and fibroblasts, remain incompletely defined, and the downstream signaling pathways through which Galectin-3 transduces injury or repair signals in the kidney remain poorly characterized. Its broad elevation in diverse inflammatory and fibrotic disorders also raises concerns about diagnostic specificity. Moreover, temporal dynamics across the entire AKI trajectory have not been fully characterized, and even in *Liu et al*.'s study, correlations between Galectin-3 and kidney function parameters, while statistically significant, were modest and less robust than those seen for creatinine or eGFR^4^. Second, the study focused on acute timepoints, leaving long-term consequences of KLF4 or Galectin-3 inhibition on epithelial repair, fibrosis, or future susceptibility unresolved. Additionally, although pharmacologic tools from *Liu et al*.'s study provided persuasive proof-of-concept, potential off-target effects merit careful evaluation. Thus, while mechanistically rich, Galectin-3's suitability as a standalone clinical biomarker remains to be fully established.

Despite these open questions, *Liu et al*. delivered an integrative and compelling narrative that positions Galectin-3 as both an injury mediator driver and a potential diagnostic marker. By situating the KLF4/Galectin-3 axis at the core of tubular apoptosis and kidney inflammation, their work illuminated the molecular choreography of early tubular injury and introduced an attractive and actionable therapeutic target. More broadly, the study exemplifies how dual-function molecules, those that both participate in injury and report it, may reshape AKI classification and management. As the field moves toward mechanism-based diagnostics and interventions, the depth and rigor of this work make it an important step toward reimagining AKI biology and may ultimately set the stage for mechanism-guided diagnostics that better align molecular injury programs with clinical decision-making.
